# Role of inflammation and oxidative stress in post-menopausal oestrogen-dependent breast cancer

**DOI:** 10.1111/jcmm.12413

**Published:** 2014-10-22

**Authors:** Clelia Madeddu, Giulia Gramignano, Carlo Floris, Giuseppe Murenu, Giuseppe Sollai, Antonio Macciò

**Affiliations:** a Department of Medical Sciences “Mario Aresu”, University of CagliariCagliari, Italy; b Medical Oncology Unit, San Gavino HospitalSan Gavino, Italy; c Department of Medical Oncology, Nuova Casa di CuraCagliari, Italy; d Department of Experimental Surgery, “A. Businco” Oncological HospitalCagliari, Italy; e Department of Oncological Surgery, “A. Businco” Oncological HospitalCagliari, Italy; f Department of Gynecologic Oncology, “A. Businco” Oncological HospitalCagliari, Italy

**Keywords:** oestrogen-dependent breast cancer, leptin, adiposity, Interleukin-6, oxidative stress

## Abstract

Weight gain and obesity are among the most important risk factors for post-menopausal oestrogen-dependent breast cancer (EDBC). Weight gain is associated with oxidative stress, which in turn promotes breast cancer progression. We carried out a prospective study in 216 consecutive post-menopausal breast cancer patients aiming to examine the correlations between traditional prognostic factors (tumour size, T, nodal, N, grading, G, and metastasis status, M), and body mass index (BMI), leptin, pro-inflammatory cytokines (Interleukin, IL,-6 and tumour necrosis factor-alpha, TNF-α), and oxidative stress (reactive oxygen species, ROS, glutathione peroxidase, GPx, superoxide dismutase, SOD) among patients with oestrogen receptor (ER)+ and ER− breast cancers. Distribution of T, N and M categories did not differ between ER+ and ER− breast cancer patients. ER− patients showed a higher incidence of G3 tumours. Weight, BMI, leptin, IL-6 and ROS were higher in ER+ compared with ER− patients. Among ER+ patients, BMI, leptin, IL-6 and ROS correlated with T and M. Leptin, IL-6 and ROS were positively correlated also with N. Among ER− patients, BMI and leptin did not correlate with any of prognostic parameters, whereas a positive correlation between IL-6, ROS and M was found. Multivariate regression analysis showed that BMI, leptin, IL-6 and ROS were predictive for T, N and M in ER+ patients. Weight gain, inflammation and oxidative stress are involved in EDBC prognosis. Their modulation through antidiabetic, anti-inflammatory and antioxidants drugs combined with endocrine therapy may constitute a targeted approach in post-menopausal EDBC.

## Introduction

Weight gain and obesity are among the most important factors that predict risk for oestrogen-dependent breast cancer (EDBC) in post-menopausal women [1]. Several pieces of evidence explain this association. (*i*) The levels of circulating oestrogens derived from peripheral aromatization of androgens are higher in overweight women than in slim post-menopausal women. (*ii*) Adiposity results in higher circulating levels of insulin and insulin-like growth factor (IGF), which stimulate growth of epithelial breast cells and induce neoplastic transformation. (*iii*) Obesity is associated with lower levels of sex-hormone binding globulin (SHBG), which correlate with increased bioavailability of circulating oestrogens [2].

More recently, it has been shown that adipose tissue is an active endocrine-immune organ that secretes adipokines [3], inflammatory cytokines and polypeptides that promote EDBC [4]. The plasma concentrations of leptin, tumour necrosis factor (TNF)-α and interleukin-6 (IL-6) positively correlate with body mass index (BMI) in overweight and obese women [4]. The increased levels of leptin and pro-inflammatory cytokines are directly associated with breast cancer development [5]. As a result of inflammatory and metabolic changes, weight gain is also associated with oxidative stress [6]. Oxidative stress damages lipids, proteins and nucleic acids [7,8], and induces activation of Akt/PI3K/mTOR signalling, promoting oncogenesis, and tumour progression, in EDBC [9,10].

The aim of this prospective observational study was to examine in a population of post-menopausal breast cancer patients the correlations between traditional prognostic factors, and BMI, leptin, circulating levels of pro-inflammatory cytokines (IL-6, TNF-α), and oxidative stress (reactive oxygen species, ROS, glutathione peroxidase, GPx, superoxide dismutase, SOD). Correlations were examined among patients with oestrogen receptor (ER)+ and among patients with ER− breast cancers. Traditional prognostic factors included grade (G), tumour size (T), node (N) and metastases (M) status according to tumour prognostic categories (TNM) staging.

## Patients and methods

Two hundred and sixteen consecutive breast cancer patients referred to the Medical Oncology Units at the ‘N.S. Bonaria’ Hospital in San Gavino, at the ‘Nuova Casa di Cura’ in Decimomannu and at the ‘A. Businco’ Hospital, Cagliari, Italy, were enrolled in the study between June 2009 and September 2013. Eligible patients were post-menopausal and had a histologically confirmed diagnosis of breast cancer. ER positivity was assigned if more than 1% of tumour cells stained positive for the oestrogen receptor. Exclusion criteria were a prior malignancy, active use of exogenous hormones (hormone-replacement therapy) at the time of blood donation, and acute and serious coexisting medical conditions, including infections. The research protocol was approved by the Institutional Ethics Committee. All subjects signed an informed written consent and participated in the study as volunteers. The study was performed in accordance with the Declaration of Helsinki.

### Measurement

Tumour histology, G, T, N and ER status were obtained from pathology reports. Tumour, N and M status were staged according to the TNM staging system. Hormone receptors were assessed by immunohistochemical assays on a selected tumour block after final surgical excision. ER was considered positive if there was >1% nuclear staining, as stated in the most recent guidelines from the American Society of Clinical Oncology and the College of American Pathologists for immunohistochemical analysis of ER in breast cancer [11]. Baseline anthropometric measurements and blood collection were performed at the time of surgery and before any systemic treatment. Weight was measured with a balance-beam scale after a 12-hr overnight fast with patients clothed in a hospital gown. BMI was calculated as weight (kg)/height (m^2^). Fasting blood (10 ml) was collected in the morning (between 8 and 9 a.m.) and placed in heparinized tubes. Samples were immediately centrifuged, and serum was stored at −70°C without thawing before performing the leptin and pro-inflammatory cytokine assays. Oxidative stress parameters were measured in fresh blood samples.

### Assessment of serum leptin and pro-inflammatory cytokine levels

Serum leptin levels were measured by a double-antibody ‘sandwich’ ELISA test (DRG Instruments, Marburg, Germany). The absorbance was measured at 450 ± 10 nm. The intra-assay and interassay variations were 5% and 7% respectively. The results were expressed in nanograms per millilitre (ng/ml). Pro-inflammatory cytokines IL-6 and TNF-α were detected in duplicate by a ‘sandwich’ ELISA with commercially available kits (DRG Instruments GmbH, Marburg, Germany). The absorbance was measured at 450 nm with a spectrophotometer. Intra-assay variations were 3% for IL-6 and 6% for TNF-α. Interassay coefficients of variation were less than 5% for both cytokines. The results were expressed in picograms per millilitre (pg/ml).

### Assessment of blood levels of ROS and antioxidant enzymes GPx and SOD

Blood levels of ROS were determined with the FORT test (Callegari, Parma, Italy). Results were expressed as FORT U (1 FORT U corresponds to 0.26 mg/l H_2_O_2_). Erythrocyte GPx and SOD activities were measured with a commercially available kit (Ransod; Randox Lab, Crumlin, UK). Results were expressed in U/L for GPx and U/mL for SOD.

### Statistical analysis

Considering a probability of 99% to detect a relationship between the independent and dependent variables at a two-sided significance level of 0.01 and a change in the dependent variables of 5 units per unit change of the independent variable (with a standard deviation of 10 and 1 respectively), a sample size of at least 200 patients should be enrolled. Descriptive means and standard deviations were generated for all study variables. The distribution of the continuous variables was checked for linearity. Differences in the anthropometrics, clinical characteristics and laboratory parameters of ER+ in comparison to ER− cancer patients were tested by the Student's *t*-test for continuous variables or the chi-squared test for categorical variables.

After checking the linearity of the data distribution and the variability among the ER+ and ER− groups, the differences in the mean levels of BMI, leptin, pro-inflammatory cytokines (IL-6 and TNF-α) and oxidative stress parameters (ROS, GPx and SOD) were compared across categories of tumour prognostic factors (pT, pN, G and M status) by one-way anova. Spearman rank correlation coefficients were calculated to examine cross-sectional interrelationships between adiposity (BMI and leptin), pro-inflammatory cytokines (IL-6 and TNF-α) and oxidative stress parameters (ROS, GPx and SOD). Then, correlations between BMI, leptin, pro-inflammatory cytokines and oxidative stress parameters and tumour prognostic factors (T, N, G and M status) were tested by Spearman's correlation test. Significant correlations were tested by multivariate regression analyses to evaluate the predictive roles of BMI, leptin, pro-inflammatory cytokines (IL-6 and TNF-α) and oxidative stress parameters (ROS, GPx and SOD) *versus* tumour prognostic characteristics. Results were considered significant for *P* < 0.01. All p values are two-tailed. Statistical analyses were performed with SPSS, version 15.0 (SPSS Inc., Chicago, IL, USA).

## Results

Two hundred and sixteen consecutive post-menopausal breast cancer patients were enrolled: 151 were ER+ and 65 were ER−. The baseline clinical characteristics of the patients are reported in Table 1. Patient distributions across tumour categories (T, N, M) did not significantly differ between ER+ and ER− breast cancer patients. Notably, G distribution and Ki-67 mean expression were significantly different between ER+ and ER−: in fact, ER− showed a higher incidence of G3 tumours and a mean higher value of Ki-67 expression. The percentage of patients with type 2 diabetes at diagnosis was higher for ER+ patients compared to that in ER−.

**Table 1:** Baseline clinical and tumour characteristics of breast cancer patients

Parameters	ER+ breast cancer patients *N* (%)	ER− breast cancer patients *N* (%)	*P*-value
Enrolled patients	151	65	
Age, years (mean ± SD)	65.8 ± 5.4	64.8 ± 8.5	0.877
Weight, kg (mean ± SD)	63.9 ± 9.3	59.1 ± 9	**<0.001**
Height, cm (mean ± SD)	158.9 ± 8.7	161 ± 6.4	0.454
History of diabetes			
Yes	98 (65)	7 (10.7)	**<0.001**
No	53 (35)	58 (89.3)	
Smoking status			
Never smoker	59 (39)	25 (39)	0.992
Former smoker	36 (24)	16 (25)	
Current smoker	56 (37)	24 (36)	
Tumour histology			
Ductal	136 (90)	63 (97)	0.078
Lobular	18 (10)	2 (3)	
Tumour size (T)			
T1	97 (64)	22 (51)	1.000
T2	35 (23)	18 (37)	
T3	12 (8)	15 (6)	
T4	7 (5)	10 (6)	
Grading (G)			
G1	43 (28)	3 (4)	**<0.001**
G2	72 (48)	14 (21)	
G3	36 (24)	48 (75)	
Nodal status (N)			
N0	95 (63)	37 (57)	0.780
N1	41 (27)	20 (31)	
N2	9 (6)	6 (10)	
N3	6 (4)	1 (2)	
Stage of disease (TNM)			
I	79 (52)	25 (39)	0.434
II	47 (31)	26 (40)	
III	18 (12)	10 (15)	
IV	7 (5)	4 (6)	
HER positive	18(12)	5 (8)	0.522
Ki67 (%)	23 ± 13.4	47.3 ± 6	**<0.001**

SD, standard deviation. Results were considered significant for *P* ≤ 0.05 as calculated by Student's t-test or chi-squared as appropriate.

### Evaluation of BMI, leptin, pro-inflammatory cytokines and oxidative stress parameters according to ER status in post-menopausal breast cancer patients

**Fig. 1:**
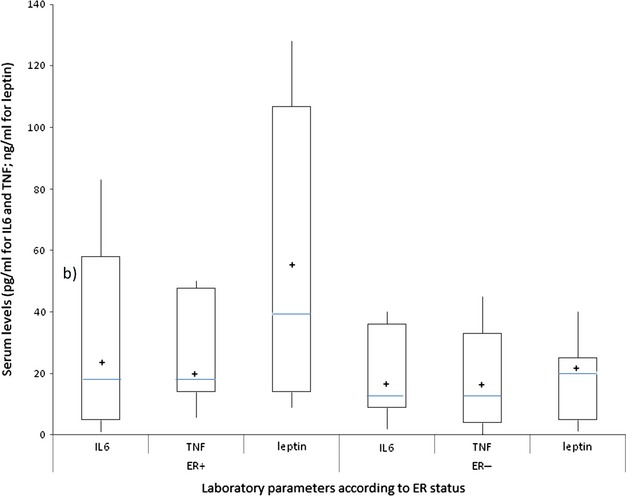
Serum levels of IL-6, TNF-α and leptin in post-menopausal breast cancer patients according to ER status. Patients with ER+ breast cancer showed significantly higher levels of IL-6 and leptin in comparison with ER− breast cancer patients. The box plots in the figure represent columns of data as boxes whose extents indicate the 25th and 75th percentiles of the column. The line inside the box represents the median. + marks the value of the mean. Capped bars indicate the minimum and maximum value observed. IL, Interleukin; TNF, Tumour necrosis Factor; ER, oestrogen receptor.

**Fig. 2:**
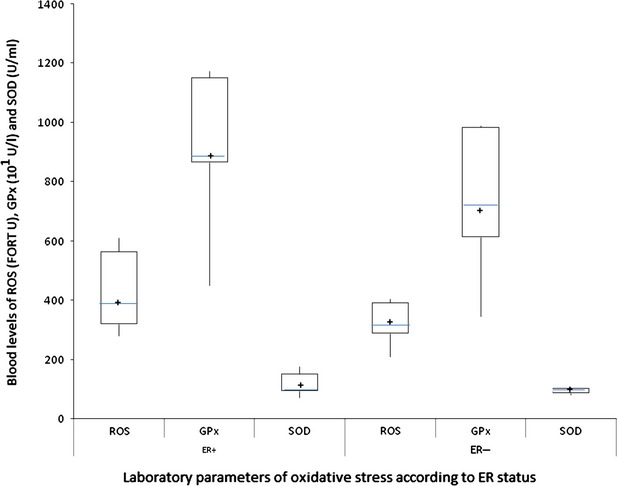
Blood levels of oxidative stress parameters (ROS, GPx and SOD) in post-menopausal breast cancer patients according to ER status. Patients with ER+ breast cancer showed significantly higher levels of ROS in comparison with ER− breast cancer patients. The box plots in the figure represent columns of data as boxes whose extents indicate the 25th and 75th percentiles of the column. The line inside the box represents the median. + marks the value of the mean. Capped bars indicate the minimum and maximum value observed. ROS, reactive oxygen species; GPx, glutathione peroxidase; SOD, superoxide dismutase; ER, oestrogen receptor.

ER+ patients showed significantly higher BMI and leptin levels in comparison with that of ER− breast cancer patients. As for pro-inflammatory cytokines, IL-6 was significantly higher in ER+, whereas TNF-α was not significantly different in the two groups of patients studied (Table 2 and Fig. 1). The levels of ROS were significantly higher in ER+ patients in comparison to those in ER− breast cancer patients. GPx and SOD were not significantly different between the two patient groups (Table 2 and Fig. 2).

**Table 2:** Baseline values of BMI, leptin, pro-inflammatory cytokines and oxidative stress parameters in post-menopausal breast cancer patients according to ER status

Parameters	ER+ breast cancer patients No. 151	ER− breast cancer patients No. 65	*P*-value
BMI, kg/m^2^ (mean ± SD)	26.7 ± 4.6	24.6 ± 3.9	**0.035**
BMI categories			
<18.5	2	4	**0.027**
18.5–24.9	49	58	
25–29.9	26	27	
>30	21	6	
Leptin, ng/ml (mean ± SD)	53.1 ± 38	24.2 ± 14.2	**<0.001**
IL-6, pg/ml (mean ± SD)	28.2 ± 20.4	16.5 ± 10.6	**<0.001**
TNF-α, ng/ml (mean ± SD)	19.4 ± 10.7	16.2 ± 14.4	0.115
ROS, FORT U (mean ± SD)	385 ± 74	325 ± 58	**0.002**
GPx, U/ml (mean ± SD)	8396 ± 2750	7542 ± 1461	0.120
SOD, U/l (mean ± SD)	110 ± 39	91 ± 57	0.067

ER, oestrogen receptor; BMI, body mass index; IL, interleukin; TNF, tumour necrosis factor***;*** ROS, Reactive oxygen species; GPx, glutathione peroxidase; SOD, superoxide dismutase.

### Evaluation of BMI, leptin, pro-inflammatory cytokines and oxidative stress parameters across TNM within ER+ and ER− breast cancer patients

Among ER+ patients, BMI and serum leptin levels were significantly higher in those patients with higher values for T, N and M+ status. Among ER− patients, no differences in BMI or serum leptin levels were observed across the different prognostic categories (Table 3).

**Table 3:** Evaluation of BMI, leptin and pro-inflammatory cytokines across tumour categories among ER+ and ER− post-menopausal breast cancer patients

Tumour characteristics	ER+ breast cancer patients				ER− breast cancer patients			
		
	BMI (kg/m^2^)	Leptin (ng/ml)	IL-6 (pg/ml)	TNF-α (pg/ml)	BMI (kg/m^2^)	Leptin (ng/ml)	IL-6 (pg/ml)	TNF-α (pg/ml)
Tumour size (T)								
T1	23.1 ± 5.7	13.9 ± 4.6	6.7 ± 1.8	17.5 ± 2.3	24.1 ± 2.6	16.1 ± 5.7	6.7 ± 2.6	21.8 ± 15
T2	23.8 ± 2.3	33.9 ± 17.3	9.2 ± 3	21.7 ± 11.6	23.9 ± 4.1	21.5 ± 4.1	18.1 ± 11.9	19.8 ± 6.7
T3	24.9 ± 4	39.6 ± 50	20.2 ± 5.2	17 ± 11.2	25.1 ± 5.4	23 ± 19.7	23 ± 19.7	21.7 ± 10.7
T4	26.5 ± 5.2*	47.8 ± 31.7*	23.9 ± 7.9*	21 ± 17.8	23.7 ± 6.9	24.6 ± 12.8	18.8 ± 12.8	27.2 ± 6.8
Tumour grade(G)								
G1	24.4 ± 4.1	29.9 ± 19.7	22 ± 10	20.1 ± 11.5	21.7 ± 4.7	16.7 ± 15	19.7 ± 5	17.9 ± 7.6
G2	23.5 ± 2.7	21.5 ± 18.4	16 ± 14	15.5 ± 3.2	22.4 ± 5.6	21.7 ± 12.1	13.8 ± 8.8	11.4 ± 8.8
G3	27.7 ± 3.5	37.3 ± 26.9	36 ± 16	22.7 ± 15.3	21.8 ± 5.8	17 ± 13.8	17 ± 4.6	11.9 ± 4.6
Nodal status (N)								
N0	24.1 ± 3.2	21 ± 16.6	7 ± 3.2	19 ± 7.8	22.9 ± 3	17.4 ± 5.0	7.9 ± 3.7	19.4 ± 12
N1	23.6 ± 4.5	35 ± 25.9	29.6 ± 17.8	16 ± 10.3	23.8 ± 5.7	24.6 ± 17.5	19.3 ± 13	18 ± 11.8
N2	25.5 ± 3.6	44.6 ± 33	35.9 ± 19.6	20.9 ± 13.2	24.9 ± 5.7	21 ± 7.3	23 ± 15	23 ± 13
N3	27.8 ± 4.3*	50 ± 22.3*	34.4 ± 21*	28.1 ± 19.2	25.3 ± 4.2	18 ± 10.4	16 ± 4.1	21 ± 9.5
Metastases status (M)								
M−	23.1 ± 3.1	17.4 ± 9	11.8 ± 9.5	18 ± 4.2	23.7 ± 3.7	21.3 ± 6.1	10.2 ± 3.4	19 ± 2.6
M+	28 ± 3.4*	64.7 ± 26.7*	49.3 ± 12*	25.3 ± 11	24.3 ± 4.7	24 ± 20	19 ± 13*	23 ± 11

* *P* < 0.05 as calculated by anova test.

BMI, body mass index; IL, interleukin; TNF, tumour necrosis factor.

Among ER+ patients relativelly pro-inflammatori cytokines only the IL-6 was significantly higher in those who had a higher tumour size, nodal involvement (N+) and distant metastases (M+). No significant differences of IL-6 serum levels were observed in ER− patients as for the prognostic categories T and N; *vice versa*, IL-6 levels were significantly higher only in M+ patients in comparison with those patients who showed no evidence of distant metastases (Table 3).

Among ER+ patients, ROS levels were significantly higher in patients with higher T, N and M+ status (Table 4). Notably, among ER− patients, ROS levels were significantly higher only in patients with distant metastases (M+) in comparison with those patients who showed no evidence of distant metastases. Among both ER+ and ER− patients, GPx and SOD levels were not significantly different according to the T, N and M status (Table 4).

**Table 4:** Evaluation of oxidative stress parameters across tumour characteristic categories among ER+ and ER− post-menopausal breast cancer patients

	ER+ breast cancer patients			ER− breast cancer patients		
		
	ROS (FORT U)	GPx (U/L)	SOD (U/ml)	ROS (FORT U)	GPx (U/L)	SOD (U/ml)
Tumour size (T)						
T1	327 ± 29	9241 ± 435	101 ± 9.1	265 ± 44	9550 ± 457	98 ± 20.3
T2	396 ± 32	9454 ± 1954	102 ± 23	337 ± 62	8942 ± 2345	90.6 ± 27
T3	405 ± 44	8416 ± 2196	112 ± 36	327 ± 105	8084 ± 2320	86.9 ± 18
T4	428 ± 99*	8735 ± 920	104 ± 29	344 ± 29	8139 ± 1494	95 ± 13
Tumour grade (G)						
G1	386 ± 74	8879 ± 1628	100 ± 22	296 ± 58	9015 ± 2353	96.6 ± 16.7
G2	368 ± 87	8839 ± 727	109 ± 24	345 ± 60	8147 ± 1567	91.2 ± 15.6
G3	410 ± 41	9747 ± 1408	115 ± 30	339 ± 48	8661 ± 1994	94 ± 14.1
Nodal status (N)						
N0	331 ± 43	9173 ± 445	102 ± 16	285 ± 45	9426 ± 1855	91.7 ± 18
N1	389 ± 27	9326 ± 1789	108 ± 35	337 ± 54	9225 ± 389	93 ± 24
N2	414 ± 139	8859 ± 22179	96 ± 17.6	318 ± 76	8396 ± 2751	90.1 ± 39
N3	429 ± 89*	7739 ± 1859	124 ± 44	380 ± 58	8700 ± 2042	89 ± 35
Metastases status (M)						
M−	351 ± 38	9318 ± 1208	108 ± 26	302 ± 21	9483 ± 165	95 ± 43
M+	460 ± 80*	8463 ± 2121	94 ± 20	450 ± 51*	8253 ± 3155	90 ± 17

* *P* < 0.05 as calculated by anova test.

ROS, reactive oxygen species; GPx, glutathione peroxidase; SOD, superoxide dismutase.

### Cross-sectional interrelationships between BMI, leptin, pro-inflammatory cytokines and oxidative stress parameters

A significant positive relationship was found between leptin and BMI in ER+ and ER− breast cancer patients. BMI and leptin correlated with IL-6 levels in ER+ post-menopausal breast cancer patients. However, no correlations were observed for BMI, leptin and pro-inflammatory cytokine levels among ER− breast cancer patients (Table 5).

**Table 5:** Cross-sectional interrelationships between BMI, leptin, pro-inflammatory cytokines and oxidative stress parameters

	BMI		Leptin (ng/ml)		IL-6 (pg/ml)		TNF-α (pg/ml)		ROS (FORT U)		GPx (UI/l)	
						
	*r*	*P*	*r*	*P*	*r*	*P*	*r*	*P*	*r*	*P*	*r*	*P*
ER+												
BMI												
Leptin	0.621	**<0.001**										
IL-6	0.502	**<0.001**	0.787	**<0.001**								
TNF-α	0.259	0.059	0.338	0.087	0.483	**0.001**						
ROS	0.339	**0.017**	0.576	**<0.001**	0.832	**<0.001**	0.263	0.074				
GPx	0.076	0.607	−0.045	0.761	−0.522	**<0.001**	−0.043	0.772	−0.343	**0.016**		
SOD	−0.017	0.907	−0.139	0.347	−0.314	**0.038**	−0.280	0.054	−0.344	**0.017**	0.285	**0.058**
ER−												
BMI												
Leptin	0.807	**<0.001**										
IL-6	−0.115	0.592	0.094	0.100								
TNF-α	0.265	0.181	−0.230	0.249	0.301	0.126						
ROS	−0.258	0.259	0.175	0.449	**0.791**	**<0.001**	0.170	0.620				
GPx	−0.115	0.592	−0.178	0.407	−0.350	0.120	−0.228	0.284	0.195	0.417		
SOD	−0.150	0.551	−0.143	0.220	−0.401	0.099	0.326	0.186	−**0.796**	**<0.001**	0.348	0.157

r, correlation factor; BMI, body mass index; IL, interleukin; TNF, tumour necrosis factor; ROS, reactive oxygen species; GPx, glutathione peroxidase; SOD, superoxide dismutase.

Reactive oxygen species levels were positively correlated with BMI, leptin and IL-6 in ER+ breast cancer patients. In addition, GPx, SOD and IL-6 negatively correlated in ER+ patients. In ER− patients, no significant correlations were found among oxidative stress parameters, BMI and leptin, whereas a positive correlation was found between ROS and IL-6 (Table 5).

### Correlations between tumour prognostic characteristics and BMI, leptin, pro-inflammatory cytokine levels and oxidative stress parameters

Among ER+ breast cancer patients, BMI, leptin and IL-6 significantly correlated with T status and presence of distant metastases (M+). Leptin and IL-6 were also positively correlated with N status (Table 6). Among ER− patients, BMI and leptin did not correlate with any of the prognostic parameters, whereas a positive correlation between IL-6 and M status was found. In ER+ patients, a significant positive correlation between ROS levels and T, N and M status was found. In ER− patients, a significant positive correlation between ROS and the presence of distant metastases (M+) was found (Table 7).

**Table 6:** Correlations of BMI, leptin and pro-inflammatory cytokines with tumour prognostic characteristics

Tumour characteristics	BMI (kg/m^2^)		Leptin (ng/ml)		IL-6 (pg/ml)		TNF-α (pg/ml)	
			
	Spearman's *r*	*P*	Spearman's *r*	*P*	Spearman's *r*	*P*	Spearman's *r*	*P*
ER+								
T	0.552	**0.038**	0.365	**0.026**	0.427	**0.002**	0.077	0.617
N	0.226	0.118	0.334	**0.019**	0.583	**<0.001**	0.152	0.320
G	0.189	0.192	0.183	0.490	0.140	0.339	0.017	0.910
M	0.325	**0.023**	0.626	**<0.001**	0.817	**<0.001**	0.128	0.128
ER−								
T	0.035	0.847	0.215	0.280	0.259	0.192	0.142	0.333
N	0.280	0.115	0.159	0.428	0.262	0.186	0.133	0.508
G	−0.219	0.220	−0.257	0.164	0.109	0.588	−0.186	0.354
M	0.028	0.876	0.296	0.134	0.449	**0.045**	0.135	0.104

r, correlation factor; BMI, body mass index; IL, interleukin; TNF, tumour necrosis factor.

**Table 7:** Correlation between oxidative stress parameters and tumour prognostic characteristics

Tumour characteristics	ROS (FORT U)		GPx (U/l)		SOD (U/ml)	
		
	Spearman's *r*	*P*	Spearman's *r*	*P*	Spearman's *r*	*P*
ER+						
T	0.355	**0.012**	−0.180	0.220	−0.092	0.534
N	0.390	**0.006**	−0.181	0.217	0.076	0.610
G	0.047	0.750	0.280	0.058	0.248	0.089
M	0.745	**<0.001**	−0.187	0.073	−0.241	0.060
ER−						
T	0.384	0.086	−0.299	0.155	−0.041	0.871
N	0.247	0.082	−0.268	0.205	−0.079	0.620
G	0.328	0.147	−0.025	0.907	−0.202	0.421
M	0.688	**0.001**	−0.121	0.414	−0.073	0.623

r, correlation factor; ROS, reactive oxygen species; GPx, glutathione peroxidase; SOD, superoxide dismutase.

The bold values correspond to significant P value.

Multivariate regression analysis showed that BMI, leptin, IL-6 and ROS were predictive for T, N and M status in ER+ patients (Table 8).

**Table 8:** Multivariate regression analysis of BMI, leptin, inflammatory and oxidative stress parameters in association with tumour prognostic characteristics among ER+ breast cancer patients

ER+ breast cancer patients	T		N		M	
		
	β coefficient	*P*	β coefficient	*P*	β coefficient	*P*
BMI (kg/m^2^)	0.729	<0.001	0.564	0.040	0.492	0.048
Leptin (ng/ml)	0.599	0.037	0.417	0.033	0.344	0.041
IL-6 (ng/ml)	0.629	0.026	1.255	<0.001	0.610	0.005
ROS (FORT U)	0.544	0.027	0.417	0.050	0.447	0.038

r, correlation factor; BMI, body mass index; IL, interleukin; ROS, reactive oxygen species.

## Discussion

In post-menopausal women, excessive weight and obesity are associated with increased oestrogen levels because of the aromatase enzyme and consequent risk of EDBC [1]. Recently, it was demonstrated that an imbalance in the production of adipokines, *i.e*. leptin, has a role in the link between weight and EDBC [12,13]. Furthermore, adiposity, especially in the context of obesity and metabolic syndrome, is associated with high levels of IL-6 and TNF-α. The roles of these inflammatory cytokines in oncogenesis and tumour progression of EDBC are well established [9].

In the present study, we confirmed that the highest BMI was linked to EDBC in post-menopausal patients [14]. Furthermore, leptin levels in ER+ patients were significantly higher than those in ER− patients. Among ER+ BMI and leptin significantly correlated with T, N and M status; then, BMI and leptin were predictive factors for the main prognostic parameters. Several studies link leptin levels with breast cancer risk [15–17]. Serum leptin, which is derived from adipose tissue in proportion to fat content, correlates with total body aromatase activity in post-menopausal patients [18]. Leptin affects several intracellular messengers that regulate proliferation and survival of breast cancer cells [19]. Notably, our results indicated that leptin specifically correlated with prognosis only among post-menopausal patients with ER+ breast cancer.

Furthermore, only in ER+ breast cancer patients, we also demonstrated a positive relationship of BMI, leptin, with IL-6. Interleukin-6 in turn was predictive of tumour T, N and M status only in ER+ patients. Pro-inflammatory cytokines may influence breast carcinogenesis through indirect weight-related effects, such as the development of insulin resistance [20,21] or the modulation of aromatase activity within adipose tissue [22]. In addition, IL-6 appears to be directly involved in the activation of NF-kB/STAT-3 [23] and insulin resistance [24]. Accordingly with this evidence, in our study several of the patients with EDBC had type-2 diabetes. Insulin resistance and elevated levels of insulin and IGF are known to induce breast cancer growth. Insulin down-regulates expression of insulin-like growth factor-binding proteins within the breast, increasing the bioavailability of IGF-1 and stimulating tumour development [25]. An indirect mechanism of action of insulin on breast carcinogenesis is the increased level of bioavailable oestrogen as a result of decreased hepatic synthesis of SHBG [26]. Moreover, insulin and IGF-1 activate the breast Akt/PI3K/mTOR pathway, which promotes cell growth and proliferation [27]. In the last years the PI3K/AKT/mTOR signalling has been found to exert a central role in the regulation of breast cancer cell growth and to modulate oestrogen receptor function. This pathway is strictly linked with several other regulatory systems for glucose-, lipid-, and aminoacid- metabolism, for energy balance and authophagy [28]. Interestingly, the Akt/PI3K/mTOR cascade mediates signalling from leptin and IL-6 [29], which in our have been shown to be significantly elevated in EDBC patients and positively correlated with tumour stage. Thus, enhanced Akt/PI3K/mTOR activation represents an interesting target for treating ER+ breast cancer [30].

Dysfunctional adipose tissue is also associated with oxidative stress, particularly in patients with obesity and type-2 diabetes mellitus [31]. The role of oxidative stress in the evolution of breast cancer has been investigated [32,33]. Post-menopausal adiposity increases the generation of free radicals, primarily through metabolic pathways [34]. Moreover, the chronic inflammation associated with adiposity may induce the production of free radicals, creating oxidative stress and promoting the development of breast cancer [35]. Based on this evidence, we evaluated whether oxidative stress was related to EDBC in post-menopausal patients.

We showed that ROS levels were significantly higher in EDBC patients and correlated with BMI, leptin, and T, N and M status. In particular, multivariate analysis showed that ROS were predictive for T, N and M status in patients with EDBC. Of note, in our study we found that also among ER− patients, ROS levels were positively correlated with IL-6, but they were both positively correlated only with M+. These findings confirm the evidence that all the metastatic diseases are characterized by the presence of chronic inflammation and associated increased ROS levels.

Consistent with our results, a central role for oxidative stress in the pathogenesis of oestrogen–induced breast cancer has been suggested. A case–control study in a wide population of breast cancer patients, including both ER+ and ER− cancers, showed that oxidative stress parameters were positively associated with breast cancer in post-menopausal women with higher BMI. Thus, the role of oxidative stress in breast cancer development may depend on adiposity [33]. More recently, a large case–control study showed that the association between increased oxidative stress and breast cancer was significant in ER+ cancers [32]. ROS are involved in oestrogen-genotoxic effects, which lead to breast cancer initiation and/or progression [36]. *In vitro* oestrogen exposure induces ROS production selectively in ER+ MCF7 cells [37,38]. Moreover, a pro-oxidative gene expression profile is associated with metastasis-free and overall survival in ER+ breast tumours [40]. It is likely that an association between oxidative stress and breast cancer prognosis may be intrinsic to an oestrogen-positive (luminal) phenotype [40]. The role of oxidative stress in ER+ breast cancer has also therapeutic implications. Increased oxidative stress in ER+ tumours seems to be associated with reduced sensitivity to conventional hormonal therapies [41]. Tamoxifen resistance in ER+ MCF-7 cells is associated with oxidative stress, increased phosphorylation of JNK and c-Jun, and increased AP-1 activity [42]. Moreover, cross-talk between the ER and PI3K/Akt/mTOR signalling has been demonstrated as a mechanism of endocrine resistance [43]. Blockade of both pathways enhances antitumour activity in pre-clinical and clinical models of breast cancer.

In conclusion, weight gain, inflammation and oxidative stress are highly involved in the pathogenesis, progression and prognosis of EDBC. Then, the selective modulation of leptin, pro-inflammatory cytokines and ROS through antidiabetic [44], anti-inflammatory [45] and antioxidants drugs [46] may constitute a new targeted therapeutic approach in post-menopausal EDBC and may potentiate the efficacy of anti-aromatase treatments in this setting of patients.
